# 152 The Role of Physical Activity and Strength Training on the Mental Health Outcomes of University Students Across the COVID-19 Pandemic: A Repeated Cross-Sectional Study

**DOI:** 10.1093/eurpub/ckae114.191

**Published:** 2024-09-26

**Authors:** Aoife Noonan, Sarah Kennedy, Catherine Norton, Audrey Tierney, Kwok Ng, Catherine Woods

**Affiliations:** Healthy UL, University of Limerick, Ireland; Health Research Institute, University of Limerick, Ireland; Physical Activity for Health Research Centre, University of Limerick, Ireland; Healthy UL, University of Limerick, Ireland; Healthy UL, University of Limerick, Ireland; Health Research Institute, University of Limerick, Ireland; Physical Activity for Health Research Centre, University of Limerick, Ireland; Sports and Human Performance Research Centre, University of Limerick, Ireland; Healthy UL, University of Limerick, Ireland; Health Research Institute, University of Limerick, Ireland; School of Allied Health, University of Limerick, Ireland; Health Research Institute, University of Limerick, Ireland; Physical Activity for Health Research Centre, University of Limerick, Ireland; Faculty of Education, University of Turku, Finland; Healthy UL, University of Limerick, Ireland; Health Research Institute, University of Limerick, Ireland; Physical Activity for Health Research Centre, University of Limerick, Ireland

## Abstract

**Purpose:**

The COVID-19 pandemic heightened mental health challenges experienced by university students. While benefits of regular moderate-to-vigorous physical activity (MVPA) and strength training physical activity (STPA) on mental health are well documented, confinements related to the pandemic restricted opportunities to engage. This paper aims to investigate associations between MVPA and STPA on the mental health and happiness of university students, prior to (2019), during (2020 & 2021) and post COVID-19 pandemic-related confinements (2022).

**Methods:**

Data from an annual repeated cross-sectional survey over 4 years (2019-2022) were analyzed. Measures included MVPA (IPAQ-SF), STPA (item from CSSPA+YA), mental health (MHI-5: dichotomized as good vs poor), happiness (single item scored from 1-10 from SAPAS) and demographic items. All measures were previously validated in the context of university students. After post-stratification weighting, independent samples t-tests and chi-square tests were conducted using IBM SPSS 29. Associations between mental health outcomes of individuals meeting weekly MVPA (150 minutes) and STPA (≥ 2 sessions) guidelines were compared to non-compliers.

**Results:**

Final participants (N = 3221) had a mean age of 21.7 (SD = 6.45) with 50.5% identifying as female. Pre-pandemic (2019), 63.4% met MVPA guidelines, compared to 50.3% (2020), 65.9% (2021) and 48.2% (2022) in subsequent years. Meeting STPA did not change from 2019 - 2021 (55% - 56%) but increased to 62% post-confinements (2022). Both happiness and mental health were statistically significantly higher pre-pandemic compared with during and post-pandemic (p-values < 0.05). Positive associations were found in all four years between meeting STPA guidelines and both mental health (p < 0.05) and happiness (p < 0.05). No associations were observed between meeting MVPA guidelines and mental health any year. Nonetheless, in 2021, meeting MVPA guidelines was associated with higher happiness scores (p = .020, g = .230). Contrary in 2022, a negative association was observed (p = 0.04, g = .119).

**Conclusion:**

STPA engagement prior to, during, and post pandemic restrictions was associated with better mental health outcomes, although associations with MVPA were inconclusive. While future research is needed, findings may be of interest to researchers and health professionals.

**Funding source:**

Healthy UL, University of Limerick.

